# PathoFact: a pipeline for the prediction of virulence factors and antimicrobial resistance genes in metagenomic data

**DOI:** 10.1186/s40168-020-00993-9

**Published:** 2021-02-17

**Authors:** Laura de Nies, Sara Lopes, Susheel Bhanu Busi, Valentina Galata, Anna Heintz-Buschart, Cedric Christian Laczny, Patrick May, Paul Wilmes

**Affiliations:** 1 Systems Ecology Research Group, Luxembourg Centre for Systems Biomedicine, Esch-sur-Alzette, Luxembourg; 2 Metagenomics Support Unit, German Centre for Integrative Biodiversity Research (iDiv) Halle-Jena-Leipzig, Leipzig, Germany; 3 Department of Soil Ecology, Helmholtz Centre for Environmental Research GmbH-UFZ, Halle (Saale), Germany; 4 Bioinformatics Core, Luxembourg Centre for Systems Biomedicine, Esch-sur-Alzette, Luxembourg

**Keywords:** Virulence factors, Bacterial toxins, Antimicrobial resistance, Mobile genetic elements, Metagenomics, Microbiome, Bioinformatics

## Abstract

**Background:**

Pathogenic microorganisms cause disease by invading, colonizing, and damaging their host. Virulence factors including bacterial toxins contribute to pathogenicity. Additionally, antimicrobial resistance genes allow pathogens to evade otherwise curative treatments. To understand causal relationships between microbiome compositions, functioning, and disease, it is essential to identify virulence factors and antimicrobial resistance genes in situ. At present, there is a clear lack of computational approaches to simultaneously identify these factors in metagenomic datasets.

**Results:**

Here, we present PathoFact, a tool for the contextualized prediction of virulence factors, bacterial toxins, and antimicrobial resistance genes with high accuracy (0.921, 0.832 and 0.979, respectively) and specificity (0.957, 0.989 and 0.994). We evaluate the performance of PathoFact on simulated metagenomic datasets and perform a comparison to two other general workflows for the analysis of metagenomic data. PathoFact outperforms all existing workflows in predicting virulence factors and toxin genes. It performs comparably to one pipeline regarding the prediction of antimicrobial resistance while outperforming the others. We further demonstrate the performance of PathoFact on three publicly available case-control metagenomic datasets representing an actual infection as well as chronic diseases in which either pathogenic potential or bacterial toxins are hypothesized to play a role. In each case, we identify virulence factors and AMR genes which differentiated between the case and control groups, thereby revealing novel gene associations with the studied diseases.

**Conclusion:**

PathoFact is an easy-to-use, modular, and reproducible pipeline for the identification of virulence factors, bacterial toxins, and antimicrobial resistance genes in metagenomic data. Additionally, our tool combines the prediction of these pathogenicity factors with the identification of mobile genetic elements. This provides further depth to the analysis by considering the genomic context of the pertinent genes. Furthermore, PathoFact’s modules for virulence factors, toxins, and antimicrobial resistance genes can be applied independently, thereby making it a flexible and versatile tool. PathoFact, its models, and databases are freely available at https://pathofact.lcsb.uni.lu.

Video abstract

**Supplementary Information:**

The online version contains supplementary material available at 10.1186/s40168-020-00993-9.

## Background

Most of the microorganisms constituting the human microbiome are commensals [1]. They contribute essential functions to the human host and contribute to its physiological development. In contrast, pathogenic microorganisms including bacteria, viruses, fungi, and protozoa cause disease by invading, colonizing, and damaging the host. Virulence factors, including bacterial toxins among others, contribute to this pathogenicity by enhancing not only the infectivity of pathogenic bacteria but also by exacerbating antimicrobial resistance which in turn restricts treatment options [1].

Virulence factors enable pathogenic microorganisms to colonize host niches ultimately resulting in tissue damage as well as local and systemic inflammation. These factors are important for pathogens to establish an infection and span a wide range, thus contributing both directly and indirectly to disease processes [2]. These virulence traits include cell-surface structures, secretion machineries, siderophores, regulators, etc. [3, 4]. However, of all virulence factors employed by pathogens, bacterial toxins often have a crucial function in the pathogenesis of infectious diseases [5]. Different types of bacterial toxins have evolved over time to counteract human defenses. These bacterial toxins can be coarsely categorized into two groups: the cell-associated endotoxins and the extracellular diffusible exotoxins. Exotoxins are typically polypeptides and proteins that act to stimulate a variety of host responses either through direct action with cell receptors or via enzymatic modulation [5, 6].

Partly through the utilization of these virulence factors, and toxins in particular, pathogenic microorganisms have been a major cause of infectious diseases including in the context of viral co-infections [1]. The development and medical use of antibiotics has limited the development and spread of these pathogens by providing an effective treatment for bacterial infections. However, the over- and mis-use of antibiotics has resulted in a global increase in antimicrobial resistance (AMR) which now threatens human health through the emergence and spread of multidrug resistant bacteria [1, 7]. As a result, many pathogenic bacteria have now acquired resistance against the main classes of antibiotics which has led to a dramatic rise in untreatable infections, resulting in the emergence of so-called “superbugs” [8]. Consequently, AMR is an urgent and growing threat to public health with an estimated number of deaths exceeding ten million annually by 2050 [9, 10].

The acquisition of antimicrobial resistance genes (ARGs) is not restricted to a single strain or species of bacteria. While commensal bacteria provide a source of ARGs, antimicrobial resistance can be transferred to pathogenic species through horizontal gene transfer, e.g., conjugation or transduction [11–13]. Therefore, to understand the emergence and spread of ARGs, it is necessary to monitor microbial communities in situ. Metagenomic sequencing, in this context, represents a pertinent technique for in situ studies as it provides less biased views of the genomic complements of individual microbial populations compared to amplicon-based methods [14, 15].

Pathogenic microorganisms have modified and adapted their virulence to host defense systems over millions of years. Similarly, AMR is thought to have evolved over extensive periods of time in bacteria, indicating that it is an ancient phenomenon [16]. However, with an increase in selective pressure through the use of antibiotics, an excessive increase in the spread and evolution of AMR has been observed in the last 50 years. Yet, despite differences in evolutionary paths, virulence factors and AMR share common characteristics. Most importantly, virulence factors and AMR are necessary for pathogenic bacteria to adapt to, and survive in, competitive microbial environments [7]. Additionally, both virulence and resistance mechanisms are frequently transferred between bacteria by horizontal gene transfer [13]. Furthermore, both processes make use of similar systems (i.e., cell wall alterations, efflux pumps, two-component systems and porins) that activate or repress the expression of various genes [17–19]. Therefore, although AMR in itself is not a virulence factor, in environments with selective antibiotic pressure, opportunistic pathogens are able to colonize through acquisition or presence of AMR [1].

Considering the burden of bacterial infections in which virulence factors and ARGs play crucial roles, it is important to be able to identify these in microbial communities. The advent of high-throughput DNA sequencing provides a powerful means to profile the full complement of DNA derived from genomic extracts obtained from a wide range of environments [20]. However, currently there is a lack of automated pipelines to simultaneously identify these different factors in metagenomic datasets. Various tools exist for the prediction of ARGs themselves, such as DeepARG [20], RGI [21], ResFinder [22], and ARGsOAP [23], with a very few prediction tools for virulence factors existing, such as MP3 [24] and VirulentPred [25]. Most of the latter tools are based on outdated databases of virulence factors which have since been expanded greatly. Moreover, there is a lack of recent bioinformatics tools for the prediction of bacterial toxin genes in particular. Furthermore, although various AMR prediction tools exist, these primarily focus on the prediction of genes without considering their location, i.e., these tools do not differentiate between localization on mobile genetic elements (MGEs) or on bacterial genomes. Since MGEs are the main mechanism by which ARGs are transmitted, it is crucial to identify the relationship between ARGs and MGEs. Outside of these prediction tools, it is common practice to use standard homology search algorithms against specific databases. However, such practices require several intermediate steps which may vary from lab to lab. Additionally, using these methods is restrictive in the sense that only a single database can be searched at a time.

Here, we present PathoFact, a pipeline for the simultaneous prediction of virulence factors, bacterial toxins in particular, and ARGs. Our tool furthermore contextualizes these with respect to their localization on MGEs. Moreover, PathoFact aggregates the information obtained via different prediction tools and databases into a single output, allowing both novices and experts in bioinformatics alike to parse information as needed. PathoFact thus provides a unified perspective on pathogenic mechanisms. We provide evaluation results on our tool’s sensitivity, specificity, and accuracy, and demonstrate PathoFact’s versality using both a simulated metagenomic dataset and public case-control metagenomic datasets for Parkinson’s disease, psoriasis, and *Clostridioides difficile* infection. Using the simulated metagenomic dataset, we further perform a comparison of PathoFact to other metagenomic characterization workflows, namely MOCAT2 [26] and HUMANn3 [27].

## Implementation

### PathoFact architecture

**Fig. 1:**
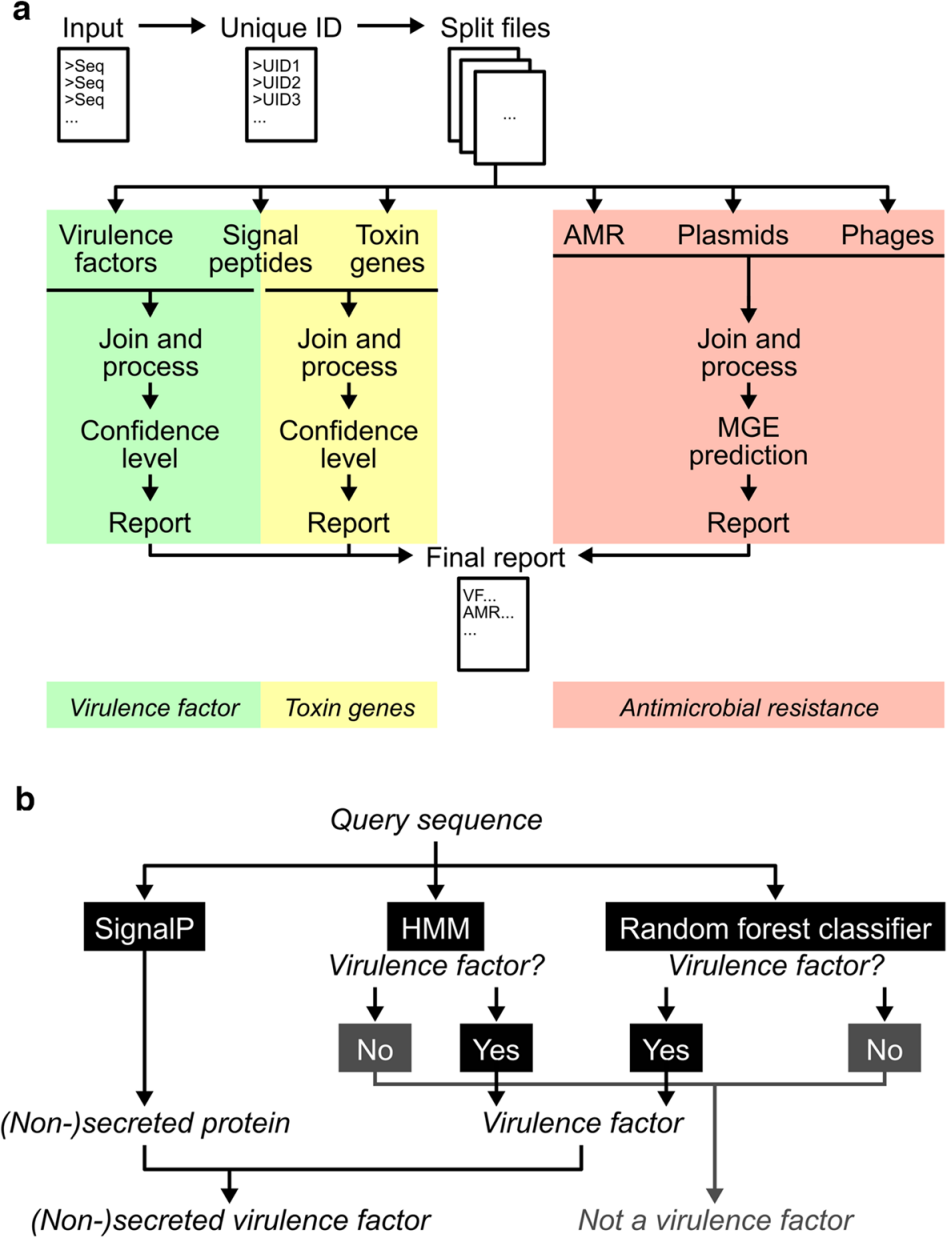
The PathoFact pipeline. **a** Framework of the PathoFact pipeline. The pipeline consists of three different modules related to (i) virulence factors, incl. (ii) bacterial toxins, and (iii) antimicrobial resistance genes. SignalP is incorporated for the prediction of secreted toxins and virulence factors. All modules can either be run independently or jointly. **b** Classification framework for the prediction of virulence factors. The prediction of virulence factors depends on two different aspects: (i) a HMM domain database, (ii) a random forest classifier. Sequences predicted positive from both are classified as virulence factors. The incorporation of SignalP in the framework allows integration of information regarding the likely secretion of the virulence factors

PathoFact is a command-line tool for UNIX-based systems that integrates three distinct workflows for the prediction of (i) virulence factors, (ii) bacterial toxins, and (iii) antimicrobial resistance genes from metagenomic data (Fig. 1a). Each workflow can be applied individually or in combination with the other workflows. Our tool is written in Python (version 3.6) and uses the Snakemake (version 5.5.4) workflow management software [28]. This implementation offers several advantages, including workflow assembly, parallelism, and the ability to resume processing following an interruption. Each step of the pipeline is implemented as a rule in the Snakemake framework specifying the input needed and the output files generated. We use conda (version 4.7) environments wherever possible thus reducing the need for explicit installation of software dependencies. Moreover, the use of conda environments makes it possible to incorporate prediction tools dependent on older Python versions incompatible with version 5.5 of Snakemake. As such, Python, Snakemake, and (mini)conda (version 4.7) [29] installations are required. PathoFact is open-source and freely available at https://pathofact.lcsb.uni.lu.

The input to the PathoFact pipeline consists of an assembly FASTA file containing nucleotide sequences of the contigs. PathoFact subsequently predicts the ORFs using Prodigal (version 2.6.3) for the prediction of virulence factors, toxins, and antimicrobial resistance genes. The MGEs are predicted from the initial assembly file, and a mapping file is generated by PathoFact which aggregates all the results. PathoFact aggregates the information obtained from the different sub modules into both module-specific reports as well as a complete final report. The reports describe all virulence factors, bacterial toxins, and antimicrobial resistance genes identified from the input as well as their assigned confidence level (virulence factors/bacterial toxins), their resistance mechanisms (AMR), and their corresponding localization on MGEs.

### Workflow for the prediction of virulence factors

For the prediction of virulence factors, we created a prediction tool consisting of two parts: (i) a database consisting of virulence factor HMM profiles (HMMER3 v3.2.1) [30] and (ii) a random forest model. Hits against the virulence factor HMM database are then combined with the classification of the random forest model to result in the final prediction (Fig. 1b). The development of the tool was inspired by the MP3 software tool for the prediction of virulence factors which has not received an update since 2014 and was thus outdated [24]. In addition, PathoFact combines these annotations with the prediction of signal peptides by SignalP (v5.0) [31] to distinguish between secreted and non-secreted virulence factors.

### Dataset for the prediction of virulence factors

A dataset, consisting of both a positive and negative subset, was constructed for the training of the virulence factor prediction tool. The positive subset consisted of known virulence factor sequences retrieved from the Virulence Factors Database (8945 sequences) (VFDB) [3]. All sequences were obtained from the VFDB core dataset containing (translated) gene sequences associated with experimentally verified virulence factors. The negative subset of the training set consisted of protein sequences that were retrieved from the Database of Essential Genes (DEG) (7995 sequences) [32] and which were known not to be virulence factors. For both subsets, all sequences were clustered with CD-HIT [33], and sequences with a 90% sequence identity were collapsed to prevent redundancy within the subsets. This 90% cutoff is routinely used to reduce redundancy in similar protein datasets, improving efficiency without foregoing specificity given the large metagenomic database sizes [34, 35]. The resulting training set was used for (i) the implementation of the HMM profiles and (ii) the training of the random forest model.

### Construction of the virulence factor HMM database

For the construction of the virulence HMM database, HMM profiles were annotated for the training set using HMMER3 (version 3.2.1) against multiple pre-compiled and in-house annotation databases [36]: PFAM-A [37], TIGR [38], KEGG [39], MetaCyc [40], and Swissprot [41]. The best hit in each HMM set was assigned to each gene in the training set if the HMM score was higher than the binary logarithm of the number of target genes, in accordance with the recommendations in the HMMer manual. HMM profiles were subsequently retrieved and the databases were concatenated to form the virulence HMM database. Binary compressed data files were constructed with the *hmmpress* (HMMER3 v3.2.1) [30]. For the prediction of virulence factors by the virulence HMM database, identified HMM profiles are separated by those matching to the positive or negative subset of the training set, as well as HMM profiles ambiguous for both positive and negative subset.

### Machine learning model for the prediction of virulence factors

In addition to the virulence HMM database, we created a random forest model [42]. A random forest model operates from decision trees and output classification of the individual trees while correcting for overfitting of the training set. While overfitting, in which models perform highly on the training set but poorly on the test set, is a common problem in machine learning, a random forest model corrects for overfitting by continuously creating trees on random subsets. This does not mean that random forest classifiers are not capable of overfitting. However, they are less sensitive to variance, and effects of overfitting are therefore rarely observed [43]. For training of the random forest model, the following five features of the sequences were selected and implemented: amino acid composition (AAC), dipeptide composition (DPC), composition (CTDC), transition (CTDT), and distribution (CTDD) [44]. A feature matrix was built with rows corresponding to the sequence composition of the features. The random forest model was implemented using pandas (v 0.25.0) [45], Numpy (v 1.17.0) [46], and scikit-learn (v0.21.3) [47] and consisted of 1600 trees with a maximum depth of 340.

### Workflow for the prediction of toxin genes

For the prediction of toxin genes, a workflow consisting of a toxin HMM database combined with SignalP version 5.0 [31] was developed. The toxin HMM database consists of bacterial toxin domains to identify toxin-related domains in the query sequences. Using the *hmmsearch* function of the HMMER3 (v3.2.1) program [30], the input query sequences are searched against the collection of profiles present in the toxin HMM database. In addition, analyses are combined with SignalP [31] to differentiate between secreted and non-secreted toxins.

### Construction of the toxin HMM database

For the toxin HMM database, an HMM model based on a training set of known toxins was developed and implemented. The training set was compiled from the Toxin and Toxin Target Database (T3DB) [48] and the training set derived from the DBETH prediction tool [5]. Protein sequences from within the training set with a similarity greater than 90% were clustered and collapsed with CD-HIT-2D to reduce redundancy [33]. The corresponding toxin HMM profiles were identified from the same five HMM databases as used for the virulence factors (see above). The datasets were extended with HMM profiles already annotated as bacterial toxin domains in the PFAM, TIGR, KEGG, MetaCyc, and Swissprot databases. Finally, in order to have a short description of all HMM profiles present in the toxin HMM database, a toxin library was created. This lists (i) all HMM profiles, (ii) their names, (iii) their alternative names, and (iv) the original database from which the HMM profile was derived.

### Workflow for the prediction of antimicrobial resistance genes

For the prediction of ARGs, the workflow is separated into two parts: (i) the prediction of ARGs and (ii) the prediction of MGEs. For the prediction of ARGs, the tools DeepARG (v1.0.1) [20] and RGI (v5.1.0) [21] are used. DeepARG uses a deep learning approach that improves classification accuracy while at the same time reducing false negatives. It offers a powerful approach for metagenomic profiling of ARGs as it expands on the available databases for ARGs by combining the widely used CARD [49], ARDB [50], and UNIPROT [51] databases. Additionally, RGI [21] is included which is able to identify mutation-driven AMR within genes, allowing for a strain-resolved profiling of AMR genes.

### MGEs: plasmids and phages

The prediction of MGEs is split into two parts focusing on the prediction of (i) plasmids and (ii) phages. For the prediction of plasmids, PlasFlow (v1.1) [52] is used, while for the prediction of phages VirSorter (v1.0.6) [53] and DeepVirFinder (v1.0) [54] were incorporated. All three tools were selected because of their performance compared to other, similar tools [52–54]. The predictions of these different tools are merged with the prediction of ARGs to provide localization information of the resistance genes to either MGEs or genomes. Considering the different predictions of MGEs, the final classification includes plasmid, phage, genome, unclassified, and ambiguous when localization predictions contradict each other, for example predicted to be both phage and plasmid.

### Evaluation of the PathoFact pipeline

To evaluate the performance of PathoFact, validations were conducted for the prediction of toxins, for virulence factors, and for ARGs. The prediction quality was evaluated by sensitivity, specificity, and accuracy criteria as defined below.

$$ \mathrm{Sensitivity}=\frac{tp}{tp+ fn}\kern3.25em \mathrm{Specificity}=\frac{tn}{tn+ fp}\kern2.75em \mathrm{Accuracy}=\frac{tp+ tn}{tp+ fn+ tn+ fp} $$

where ***tp*** represents true positives (i.e., virulence factors (incl. bacterial toxins) or AMR gene is predicted correctly), ***tn*** (i.e., a gene is correctly predicted not to be a virulence factor, toxin genes, or AMR gene), ***fp*** false positive (i.e., a gene incorrectly identified as a virulence factor, toxin genes or AMR gene), and ***fn*** false negatives (i.e, a virulence factor, toxin genes or AMR gene is incorrectly identified as non-pathogenic). We evaluated the sequence similarities between the training and validation (test set) datasets after removing the sequences from the validation set with 90% identity to the training set sequences using sourmash [55] (Additional File 1: Figure S1).

### Validation of virulence factors

A validation dataset was constructed to assess the performance of the prediction of virulence factors. Analogous to the training set, the validation set consisted of a positive subset of 2639 sequences (VFDB database) and a negative subset of 2628 (DEG database) sequences. Importantly, the sequences in the validation dataset were removed from the training set to avoid overfitting. The test set for virulence predictions was used to run both the standalone MP3 (v1.0) tool and our newly generated tool for prediction of virulence factors. For MP3, the standard advised parameters were used: set on metagenomic protein fragments, a minimum length of 90 bases and a threshold value of 0.2 for the svm module [24].

### Validation of toxin genes

For the validation of toxin genes, a validation dataset containing both positive and negative subsets was constructed. The positive subset was constructed from sequences in the EMBL-EBI database annotated as bacterial toxins. The results were limited to protein sequences described in the UniProtDB. Further filtering of the protein sequences removed sequences with uncertain predictions (i.e., hypothetical, probable). To limit redundancy within the dataset, sequences were clustered in terms of similarity by using a 90% sequence identity cutoff. Furthermore, to limit redundancy between the validation and the training set, sequences with a similarity of greater than 90% were discarded. The remaining 202 positive sequences were combined with 202 random-selected sequences from the negative dataset, consisting of housekeeping genes representing the validation dataset.

### Validation of AMR prediction

For the prediction of AMR genes, both the DeepARG and RGI prediction tools were used. DeepARG has proven to be more accurate than most AMR prediction tools with a great reduction in false negatives [20], while RGI is capable to annotate SNPs contributing to AMR. For further validation, before inclusion in the pipeline, the prediction tools were tested using the NCBI’s resistance gene database (5265 sequences) [56]. This positive subset was combined with a negative subset (consisting of sequences retrieved from the Database of Essential Genes) of equal size. For DeepARG default settings were applied, while parameters for model were set to **LS** and type was set to **prot**. Similar to DeepARG, default settings of RGI were applied while input-type was set to **protein.**

### Data analysis and data availability of publicly available datasets

Metagenomic sequences for the publicly case-control metagenomic datasets were obtained from the European Bioinformatics Institute-Sequence Read Archive database, with accession numbers PRJNA297269 (Milani et al. [57]), PRJNA281366 (Tett et al. [58]), and ERP019674 (Bedarf et al. [59]). Information on the analyzed samples per study can be found in Additional File 1: Table S1. Metagenomic reads were processed and assembled using IMP (v2) [60]. The resulting FASTA files containing the assembled contigs and genes were used as input for PathoFact. For analyses of the predictions, FeatureCounts (v1.6.4) [61] was used to extract the number of reads per functional category. Thereafter, the relative abundance of the toxin genes was calculated using the Rnum_Gi method described by Hu et al [62]. Additionally, the DESeq2 (v1.24) [63] package was used to analyze the differential abundance of virulence factors, toxins, and AMR genes.

### Data analysis and data availability of a simulated dataset

To evaluate the performance of PathoFact compared to other metagenome characterization workflows, a high-complexity stimulated dataset consisting of 5 time series samples with 596 genomes and 478 circular elements was obtained from CAMI [64]. As with the case-control metagenomic dataset reads were processed and assembled using IMP (v2), after which the dataset was run through PathoFact. In addition, both MOCAT2 and HUMAnN3 were run on the stimulated metagenomic dataset using default settings of both workflows. Further data analysis was performed as described for the case-control datasets.

## Results and discussion

### Benchmarking

The PathoFact pipeline has an in-built multi-threading option to improve computational efficiency. In fact, certain tools, e.g., DeepVirFinder, are memory intensive and may require additional resources. Table 1 corresponds to the runtime of a metagenomic dataset (363,933 metagenomic sequences) with differing numbers of threads. A minimum usage of 8 threads, in this case corresponding to 28 GB/thread, is advised for running the pipeline. Additionally, for the installation of PathoFact, an initial storage of 6.3 GB is required.

**Table 1:** PathoFact runtimes with different threads/computational resources

Threads	Memory	Running time
8	224 GB	25 h 19 min
16	448 GB	15 h 58 min

### Validation of the PathoFact pipeline

For the prediction of virulence factors, the prediction tool consists of two parts: a virulence factor HMM database and a random forest classifier. The random forest classifier’s out-of-bag (OOB) error value reported an accuracy of 0.822. To improve performance for virulence prediction, the random forest model was combined with the HMM database which resulted in an overall sensitivity of 0.886, specificity of 0.957, and an accuracy of 0.921 (Table 2). Additionally, we compared our tool to the MP3 tool for the prediction of virulence factors (Additional File 1: Table S2). PathoFact scored overall higher than MP3 which scored 0.125, 0.992, and 0.558, respectively. In addition to the prediction of virulence factors, for the prediction of bacterial toxins, an overall sensitivity of 0.777, specificity of 0.989, and accuracy of 0.832 were obtained. Finally, for the prediction of ARGs, the sensitivity, specificity, and accuracy of both DeepARG and RGI were determined at 0.720, 0.996, 0.858 and 0.920, 0.997, 0.958, respectively. A combined approach merging the use of both tools resulted in the highest scores with an overall sensitivity of 0.963, specificity of 0.994, and accuracy of 0.979 for the prediction of AMR genes.

**Table 2:** Validation of the PathoFact pipeline

	Toxin prediction	Virulence factor prediction	AMR prediction
Sensitivity	0.777	0.886	0.963
Specificity	0.989	0.957	0.994
Accuracy	0.832	0.921	0.979

### Performance evaluation using a simulated dataset

**Fig. 2:**
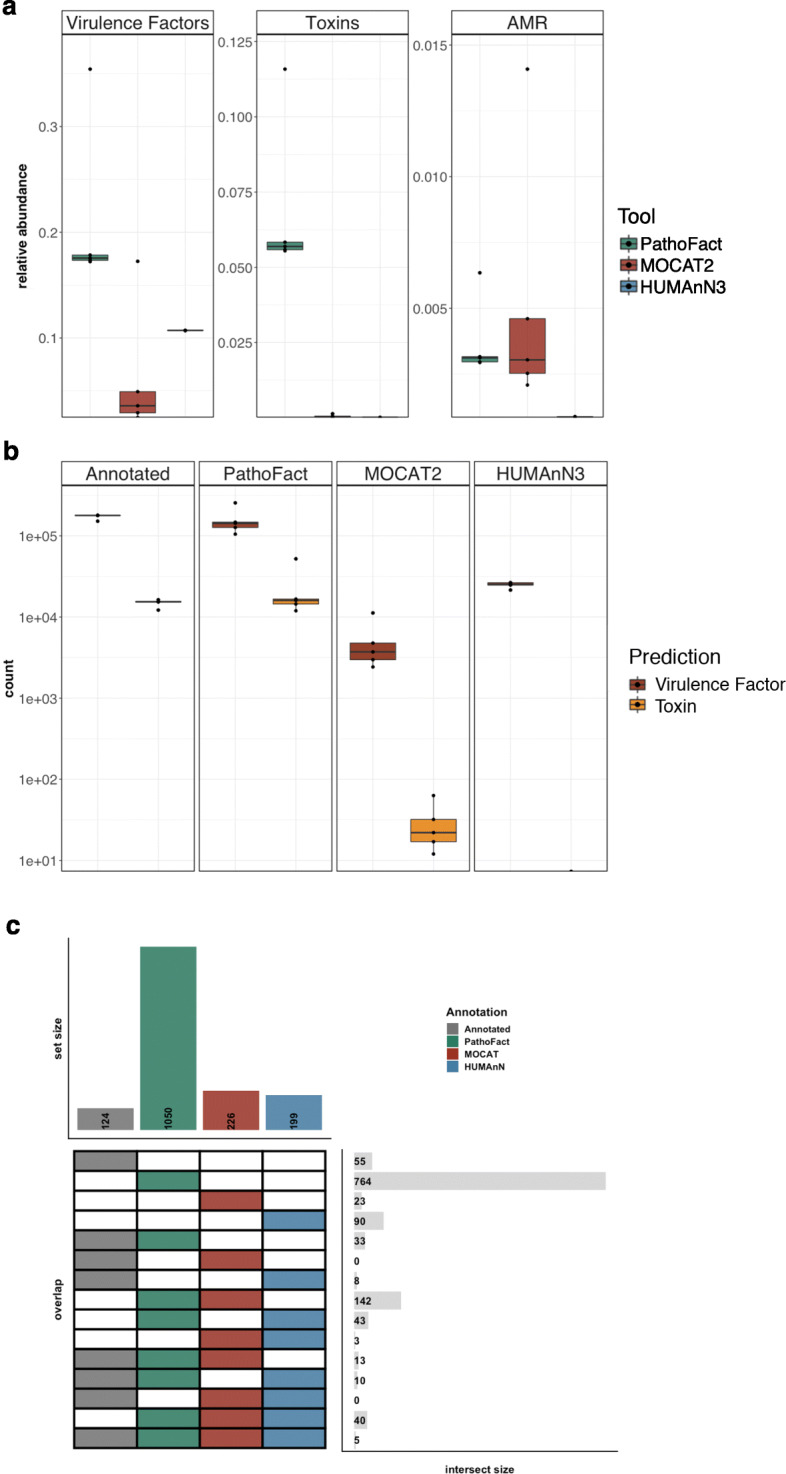
Performance evaluation of PathoFact on a high-complexity simulated dataset. **a** The relative abundances (%) of virulence factors, including bacterial toxins, as well as antimicrobial resistance, as predicted by PathoFact, MOCAT2, and HUMAnN3, * two-way ANNOVA, *P* value < 0.05. **b** Total number of virulence factors and toxin genes identified in the annotated genome and as predicted by PathoFact, MOCAT2, and HUMAnN3 **c** Number of unique ARGs as annotated by the NCBI resistance database and as predicted by PathoFact, MOCAT2, and HUMAnN3

To further evaluate the performance of PathoFact and compare it to other existing tools, the PathoFact pipeline was run on a simulated metagenome comprised of high-quality annotated genomes, i.e., the CAMI high complexity toy test dataset. Both MOCAT2 [26] and HUMAnN3 [27] were run on the original reads of the simulated CAMI datasets, while the same read datasets were processed and assembled with IMP followed by execution of PathoFact. Subsequently, annotations resulting from the different workflows were compared to evaluate the performance of PathoFact (Fig. 2a). PathoFact demonstrated increased numbers of predictions compared to both MOCAT2 and HUMAnN3 regarding virulence and toxin predictions (< 0.05, ANOVA) while performing similarly regarding AMR prediction compared to MOCAT2. Furthermore, and importantly, no additional curation or data-wrangling is needed for PathoFact compared to the other workflows tested above.

Additionally, we aimed to further characterize the performance of the metagenomic workflows against annotations of the CAMI high complexity toy test dataset. To achieve this, we annotated the underlying genomic data using the NCBI database of resistance genes [56], as well as a BLAST search of the original 450 genomes against known virulence factors and toxin genes [3, 5]. The resulting annotations were compared to the prediction reports of PathoFact, MOCAT2, and HUMAnN3. PathoFact identifies a similar number of virulence factors and toxin genes in the annotated genomes compared to the original annotations, while MOCAT2 and HUMAnN3 identified a significantly lower number (Fig. 2b). Regarding antimicrobial resistance, PathoFact was able to identify many more gene variants compared to MOCAT2 and HUMAnN3 (Fig. 2c).

### Performance of PathoFact on metagenomic datasets

Virulence factors and toxins may contribute to dysbiosis of the microbiome and favor a pro-inflammatory environment [65]. In addition, particular pathogenic bacteria may adapt to, and survive in, the presence of antimicrobials through acquisition or expression of AMR. Thereby, virulence factors, toxins, and AMR may all contribute to the pathogenic potential of the microbiome, which in turn may have an effect on the onset and development of disease and infection. The performance of PathoFact was demonstrated using three publicly available case-control metagenomic datasets which were chosen considering the following criteria: representing an actual infection or a chronic disease in which either pathogenic potential or toxins are believed to play a role. The Milani et al.’s [57] study represents actual infections with *Clostridioides difficile* (CDI) in the human gut microbiome of five patients along with five healthy controls. Furthermore, skin metagenomes of five psoriasis patients along with five healthy controls from Tett et al. [58] were chosen to represent a chronic disease in which a pathogenic potential is believed to have a function. Additionally, from Bedarf et al*.* [59], the metagenomes of fecal microbiomes derived from 10 early stage Parkinson’s disease (PD) patients, as well as 10 age-matched controls, was obtained to represent a chronic disease in which bacterial toxins are believed to be involved [59].

### Prediction of virulence factors and bacterial toxins

**Fig. 3:**
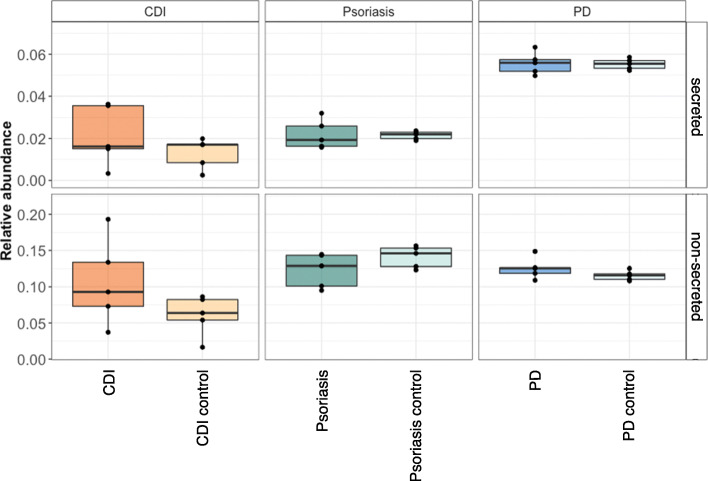
Virulence factors in three case-control metagenomic datasets. The relative abundances (%) of both secreted and non-secreted virulence factors as well as non-pathogenic sequences in three metagenomic datasets (*Clostridioides difficile* infection, Psoriasis, Parkinson’s disease)

The predictions from PathoFact resulted in the identification of virulence factors in all three case-control metagenomic datasets. Furthermore, predicted virulence factors were characterized as secreted and non-secreted through the incorporation of SignalP in the pipeline. No statistically significantly (*P* value < 0.05, Wilcoxon rank sum test) different relative abundance of the different virulence factors was found in any of the three studies when comparing diseased state and control (Fig. 3).

**Fig. 4:**
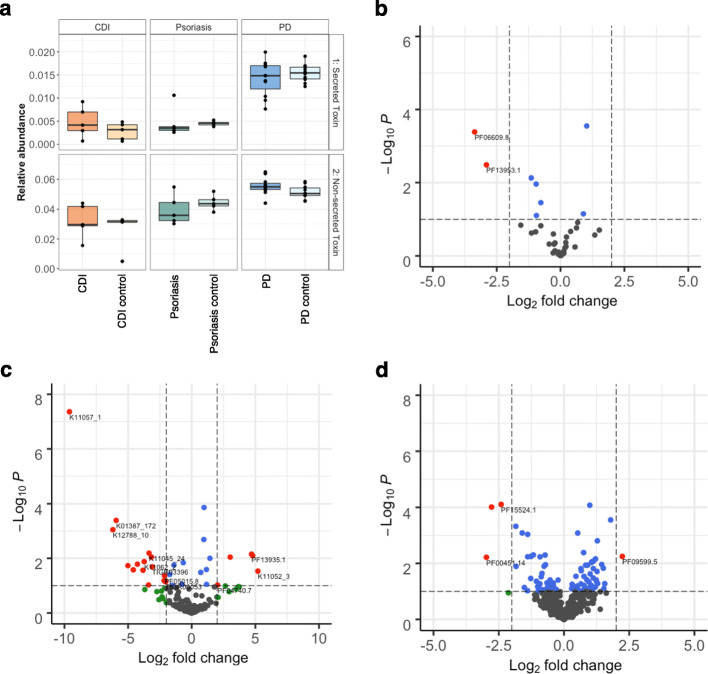
Bacterial toxins in three case-control metagenomic datasets. Bacterial toxins in disease versus control datasets. **a** The relative abundance (%) of both secreted and non-secreted bacterial toxins in diseased versus control subjects. **b** Volcano plot depicting differentially abundant bacterial toxins in *Clostridioides difficile* infections versus control. **c** Volcano plot depicting differentially abundant bacterial toxins in Psoriasis versus control. **d** Volcano plot depicting differentially abundant bacterial toxins in Parkinson’s disease versus control

In addition to the general prediction of virulence factors using PathoFact, we identified bacterial toxins, as well as their corresponding HMM domain by which they were identified. Furthermore, both secreted and non-secreted toxins were identified in both diseased and control groups in all datasets (Fig. 4a), and we identified several differentially abundant bacterial toxins (Additional File 1: Table S3-S5). Within the CDI dataset, three distinct toxin domains, PF13953, PF13954, and PF06609, were identified to be differentially abundant in CDI over control (Fig. 4b). Interestingly, none of these toxin domains have yet been reported to be linked to CDI and therefore are of interest for further research. Four distinct toxin domains (K12340, PF13935, PF14449, and K11052) were found to be significantly abundant in psoriasis over controls (Fig. 4c). Of these toxin domains, only K12340 was previously linked to psoriasis [66]. Finally, regarding the PD study we found several differentially abundant bacterial toxins when comparing PD and control samples (Fig. 4d). Of these bacterial toxins, one containing the PF09599 domains was more abundant in PD and is among others found in invasin proteins in *Salmonella typhimurium* which has been hypothesized to be involved in Parkinson’s disease [67]. Interestingly, in all three datasets additional “unknown” toxin domains were identified to be linked to the diseases, therefore representing interesting candidates for further research.

### Prediction of antimicrobial resistance

**Fig. 5:**
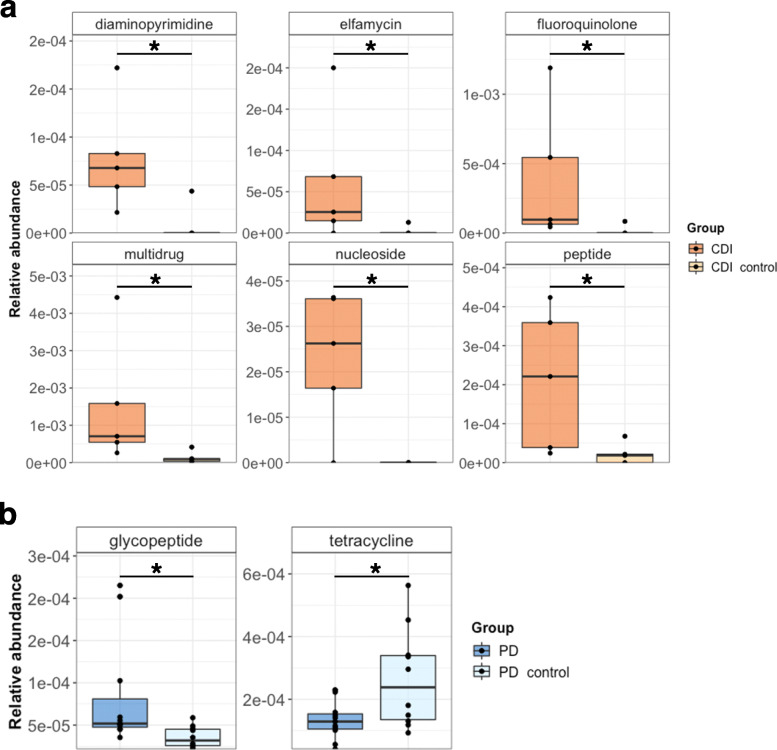
Antimicrobial resistance in three case-control metagenomic datasets. The relative abundance (%) of antimicrobial resistance categories with statistically significantly differential abundance in **a** *Clostridioides difficile* infection versus control, **b** Parkinson’s disease vs control. *P-value < 0.05

Using the PathoFact pipeline, we predicted the presence of antimicrobial resistance genes in all three case-control metagenomic datasets. Within the CDI datasets, 23 ARG categories were identified (Additional File 1: Figure S2a) of which six, i.e, diaminopyrimidine, elfamycin, fluoroquinolone, nucleoside, peptide, and multidrug, were significantly higher abundant in individuals with CDI over control (Fig. 5a). Antimicrobial resistance has previously been found to be associated with CDI infections [68]. In the metagenomic data of the skin microbiome, 22 categories of ARGs were identified (Additional File 1: Figure S2b). Interestingly, none of these resistance categories were found to be significantly different, neither with the diseased nor the control group. Within the PD study, 33 ARG categories were identified (Additional File 1: Figure S2c) with glycopeptide resistance significantly abundant in PD over controls, while tetracycline resistance was found to be enriched in the control group (Fig. 5c). The link between antimicrobial resistance and Parkinson’s disease has been mostly unexplored thus far. However, a recently published study by Mertsalmi et al. [69] suggests a role for antibiotics in PD through the influence on the gut microbiome.

Although we propose the primary usage of PathoFact for metagenomic analyses, as seen with these three case-control metagenomic datasets, it can also be applied to single genome assemblies. Using the *Klebsiella pneumoniae* subsp. Pneumoniae HS11286 reference genome, we identified 86 resistance genes of which 6 contained SNPs contributing to resistance (Additional File 1: Table S6).

### Prediction of mobile genetic elements linked to virulence factors

**Fig. 6:**
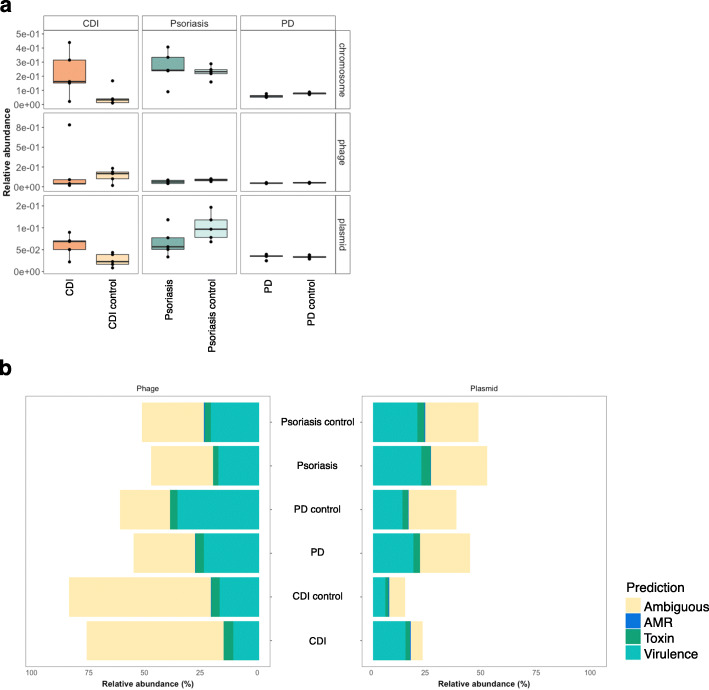
Identification of MGEs within three case-control metagenomic datasets. Relative abundance of MGEs within three metagenomic datasets (*Clostridioides difficile* infection, psoriasis (skin), and PD). **a** The overall relative abundance of phage and plasmids within the *Clostridioides difficile* infection, psoriasis, and Parkinson’s disease datasets. **b** The distribution of virulence factors, incl. toxins, and AMR between phage and plasmid in all datasets

Using the predictions generated by PathoFact, we resolved the genomic contexts and identified MGEs in all three case-control metagenomic datasets (Fig. 6a) (Additional File 1: Figure S3). Within all three datasets, the presence of both phage- and plasmid-derived sequences was detected, although no significant difference was observed between diseased and control. We found that in all datasets the majority of MGEs were found to be both linked to virulence factors as well as AMR (~ 50%), closely followed by MGEs linked solely to virulence factors, including bacterial toxins, with AMR contributing to the remaining MGEs (Fig. 6b). Furthermore, a number of MGEs were found to be both linked to virulence factors as well as AMR.

Of the ARGs linked to MGEs, the prevalence of the different resistance categories were identified using our tool. Within the CDI dataset, the majority of the MGEs were linked to phenicol and beta-resistance in both diseased and control groups (Additional File 1: Figure S4a). Additionally, plasmids linked to diaminopyrimidine and sulfonamide resistance were identified within the disease group while found to be absent in the control. Within the skin metagenomes, the majority of the predicted resistance genes linked to MGEs included beta-lactam, tetracycline, and multidrug resistance in both diseased and control groups (Additional File 1: Figure S4b). However, MGEs linked to beta-lactam resistance were found to be enriched in the diseased group. Finally, of the resistance genes within the PD study, both peptide and tetracycline resistances were found to be linked to phage and plasmids. Peptide resistance was abundant in controls whereas tetracycline was identified primarily in diseased (Additional File 1: Figure S4c).

## Conclusions

The identification of virulence factors, toxins, and antimicrobial resistance genes are of immediate importance for understanding the pathogenic state of microbiomes. Using our newly developed tool, PathoFact, we were able to identify virulence factors and bacterial toxins within three publicly available case-control metagenomic datasets. Furthermore, we were able to identify differentially abundant bacterial toxins when comparing diseased and control groups in all datasets. Additionally, antimicrobial resistance genes were identified in two of the datasets with a significant difference of certain resistance categories between diseased and control individuals. The inclusion of MGEs is of particular importance in understanding the possible transmission of MGE-born virulence factors. With PathoFact, we identified MGEs in all three datasets and were able to link these simultaneously to the corresponding virulence factors, toxins, and antimicrobial resistance genes.

Until now, no single tool has existed which has combined these distinct aspects. Although several prediction tools exist for AMR, DeepARG and RGI have been chosen for their accuracy and ability to identify mutation contribution to resistance, and were included in our pipeline. Limited or no tools were available on the other hand for the prediction of toxins and virulence factors. PathoFact utilizes the wealth of currently available software (e.g., AMR and MGE predictions) as well as newly generated tools (e.g., virulence factors and toxins). Furthermore, PathoFact can conveniently integrate updates and newly developed prediction tools. In conclusion, our tool combines the strength of AMR predictions linked to MGE predictions and integrates this with the prediction of toxins and virulence factors. PathoFact is a versatile and reproducible pipeline by its ability to run either the complete workflow or each module on its own, giving the investigator flexibility in their analysis.

## Availability and requirements

**Project name:** PathoFact

**Project home page:** https://pathofact.lcsb.uni.lu

**Operating system(s):** Platform independent

**Programming language:** python

**Other requirements:** snakemake (version > = 5.5), conda (version > = 4.7)

**License:** GNU GPLv3.

**Restrictions to use by non-academics:** see License

## Supplementary Information

**Additional file 1.** PathoFact supplementary materials.

## Data Availability

PathoFact, its models, and databases are available at https://pathofact.lcsb.uni.lu.

## Abbreviations

AMR: Antimicrobial resistance
ARG: Antimicrobial resistance gene
CDI: *Clostridioides difficile* infection
PD: Parkinson’s disease
