# Supplementary material for: PathoFact: a pipeline for the prediction of virulence factors and antimicrobial resistance genes in metagenomic data
Source: Microbiome. 2021 Feb 17;9:49. doi: 10.1186/s40168-020-00993-9 (PMC7890817; doi:10.1186/s40168-020-00993-9)
Supplement: Supplementary file 2 — Additional file 1. PathoFact supplementary materials. [file 40168_2020_993_MOESM2_ESM.pdf]

PathoFact: A pipeline for the prediction of virulence factors and antimicrobial resistance genes in metagenomic data

Laura de Nies[1], Sara Lopes[1], Susheel Bhanu Busi[1], Valentina Galata[1], Anna Heintz-Buschart[1,2,3], Cedric Christian Laczny[1], Patrick May[4], Paul Wilmes[1]

[1] Systems Ecology research group, Luxembourg Centre for Systems Biomedicine, Esch-sur-Alzette, Luxembourg
[2]Bioinformatics Unit, German Centre for Integrative Biodiversity Research (iDiv) Halle-Jena-Leipzig, Germany
[3]Department of Soil Ecology, Helmholtz Centre for Environmental Research GmbH - UFZ, Halle (Saale), Germany
[4]Bioinformatics Core, Luxembourg Centre for Systems Biomedicine, Esch-sur-Alzette, Luxembourg

Contents

List of Supplementary Figures

S1 Sequence similarity comparison between validation and training datasets. 2

S2 Antimicrobial resistance in three case-control metagenomic datasets 2

S3 Distribution of virulence factors, toxins and AMR over unclassified and ambiguous 4

S4 The prevalence of different resistance categories within the MGEs 5

List of Supplementary Tables

S1 Samples analyzed using PathoFact 6

S2 Comparison of tools for the prediction of virulence factors 7

S3 – S5 Differentially abundant bacterial toxins in all three datasets 7

S6 Antimicrobial resistance identified in Klebsiella *pneumoniae* genome 8

Supplementary Figures

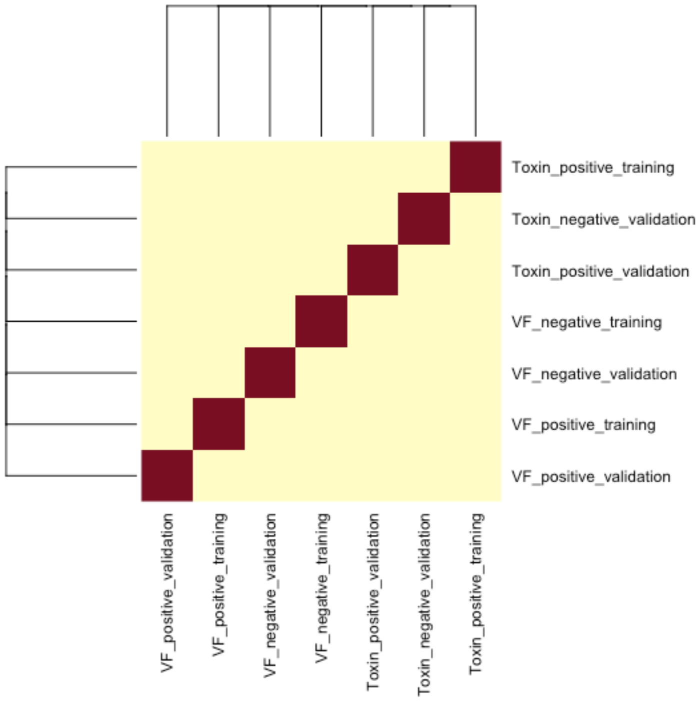

Figure S1: Sequence similarity comparison between validation and training datasets.

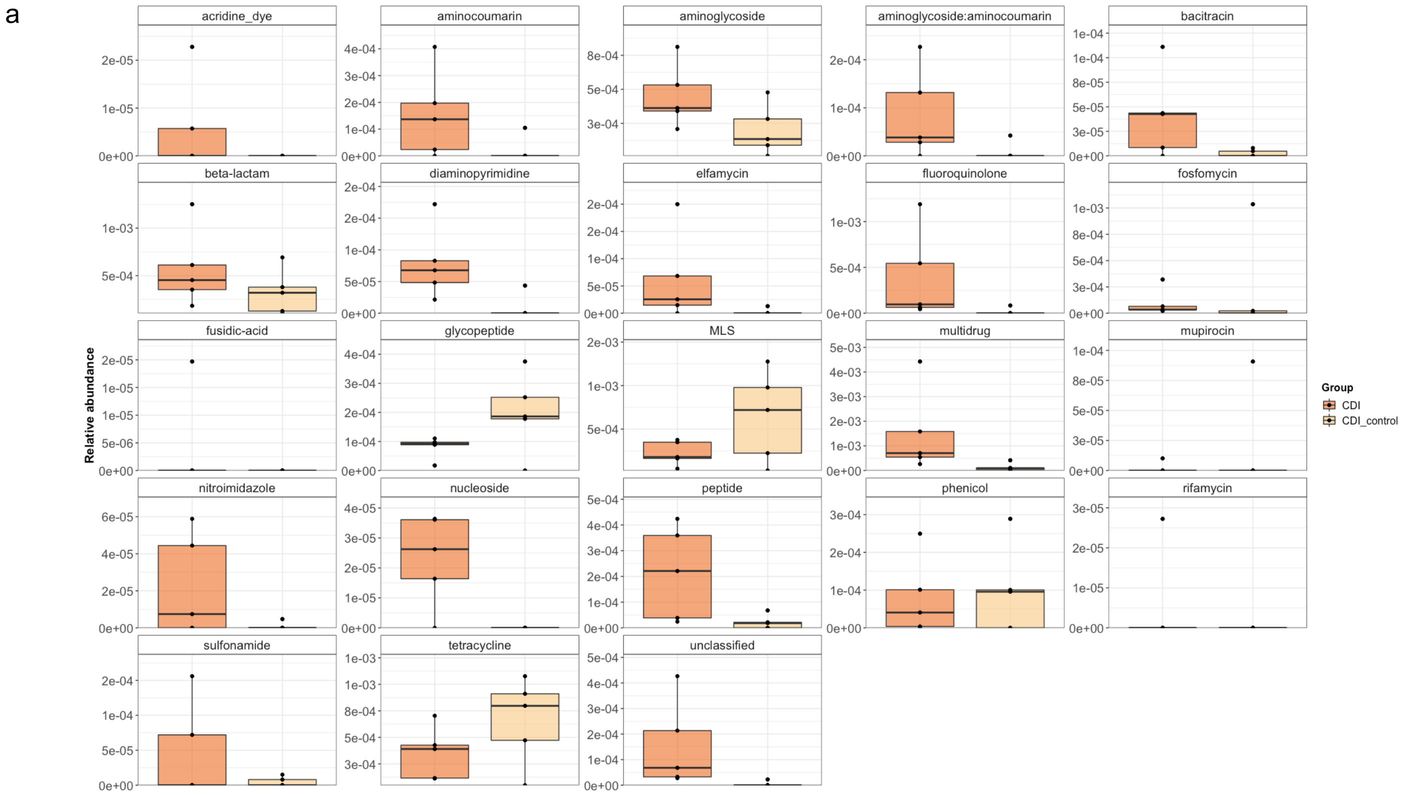

b

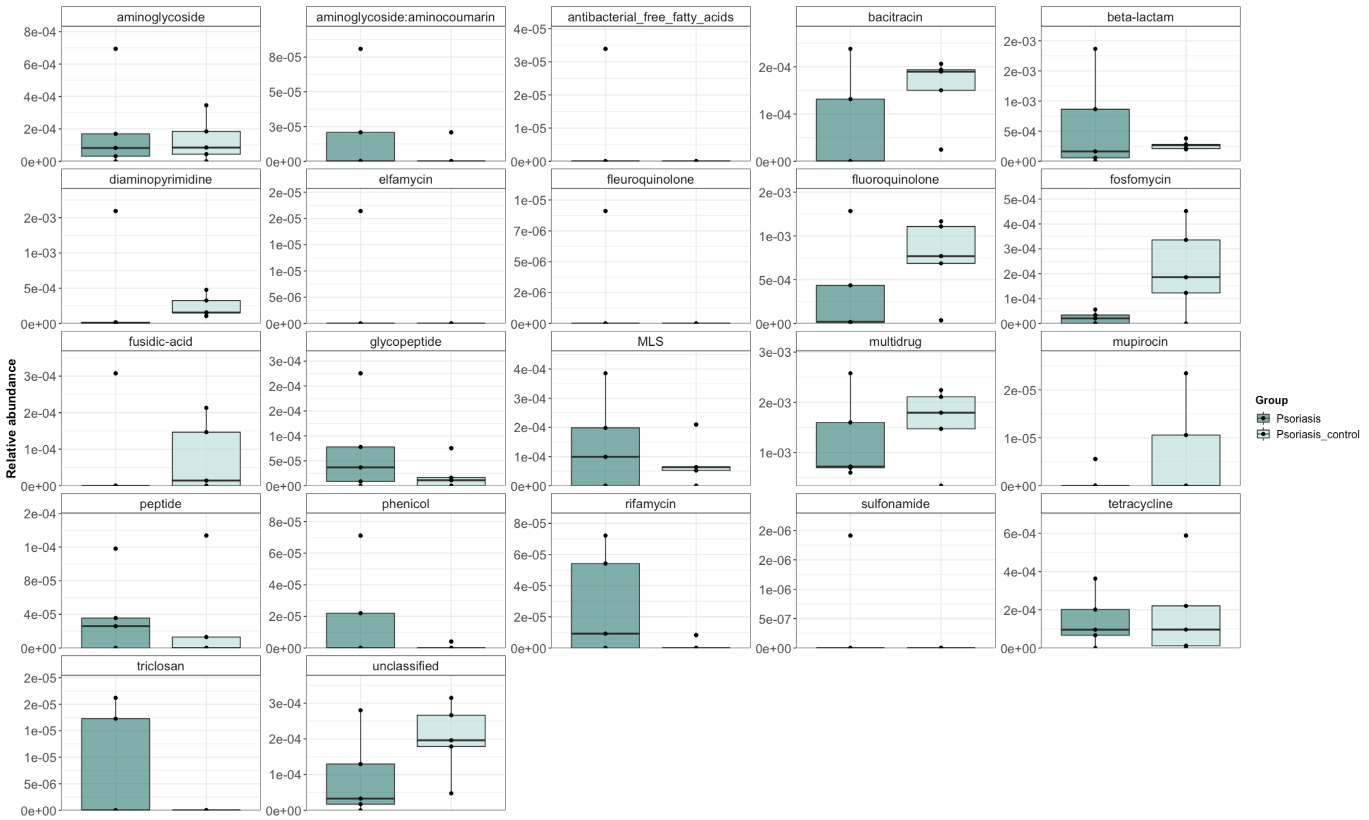

c

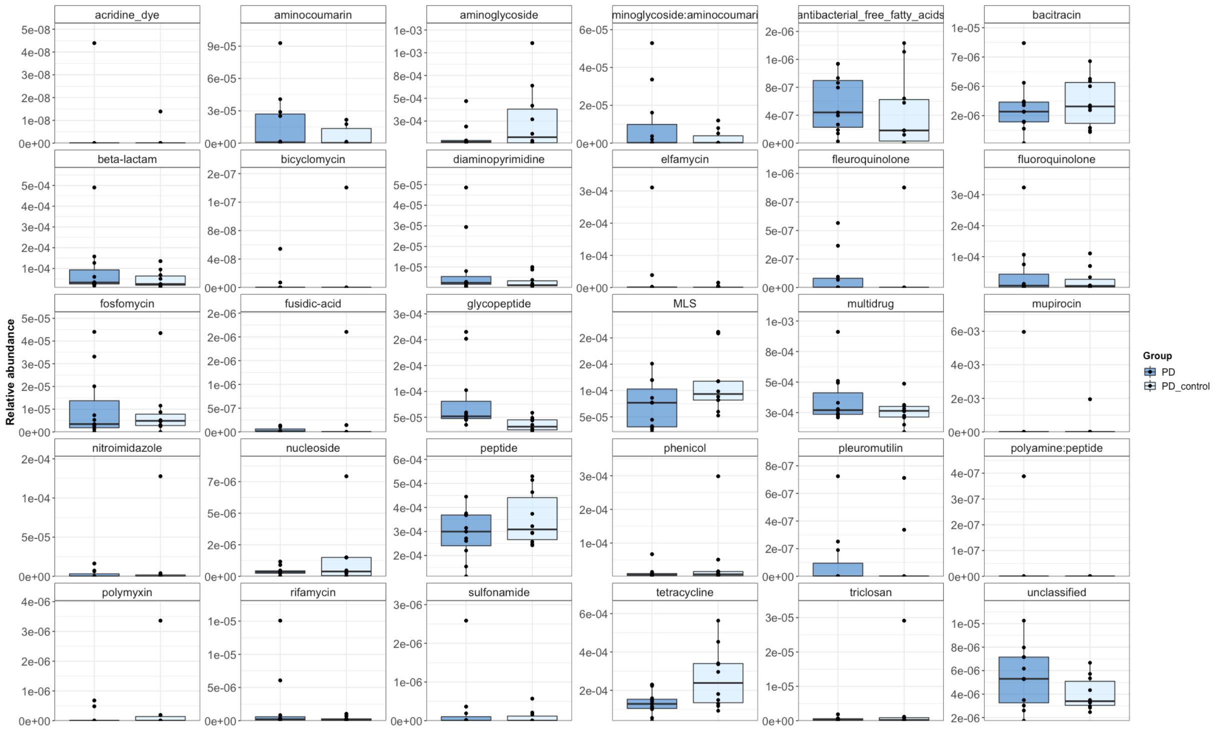

Figure S2: Relative abundance of antimicrobial resistance categories in three case-control metagenomic datasets. Relative abundance (%) of all identified resistance categories **a.** 23 antimicrobial resistance categories within *Clostridioides difficile* infection **b.** 22 antimicrobial resistance categories within the skin metagenome (psoriasis) and **c.** 30 antimicrobial resistance categories within the Parkinson’s disease study.

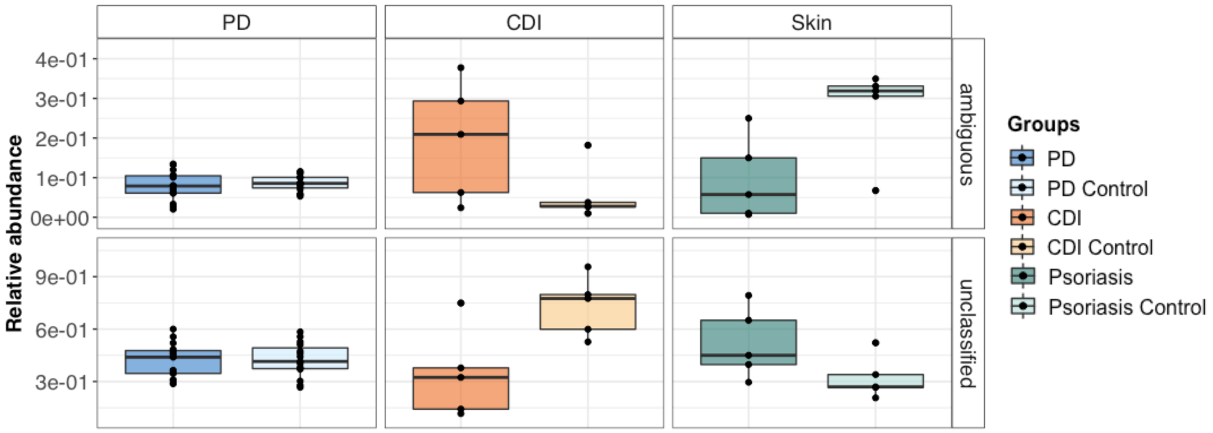

Figure S3: Distribution of virulence factors, including bacterial toxins, and AMR over unclassified and ambiguous (predicted to be both plasmid and phage or phage and chromosome)

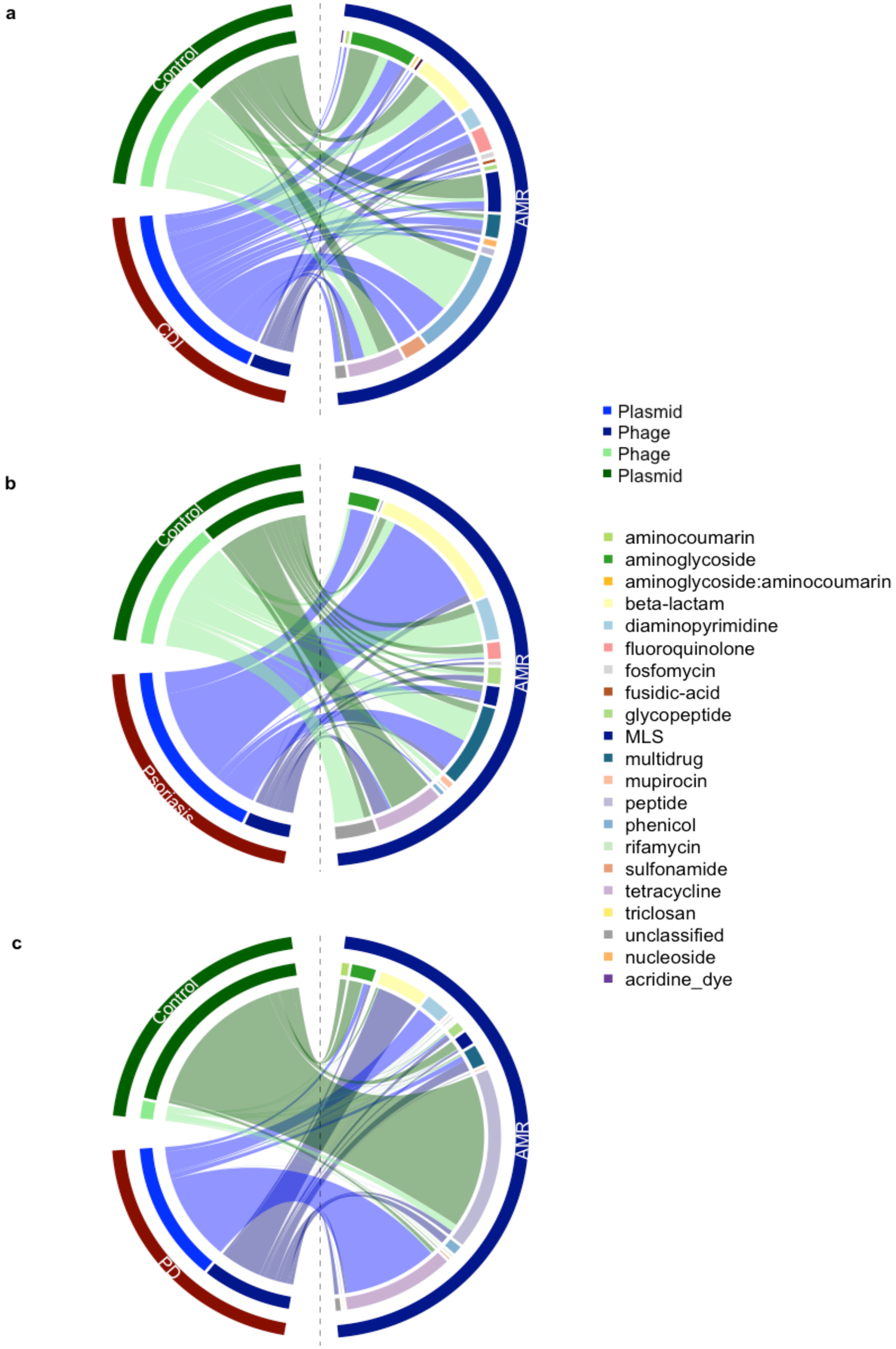

Figure S4: The prevalence of different resistance categories within the MGEs. a. prevalence of antimicrobial resistance genes within MGEs in Clostridioides difficile infection and control. b. psoriasis and control c. Parkinson's disease and control.

Supplementary Tables

Table S1: List of samples analyzed using the PathoFact pipeline grouped by originating study.

Study	Cohort	Group	Accession number	Sample
Bedarf. et al	Parkinson's disease	PD	ERP019674	ERS1647277
Bedarf. et al	Parkinson's disease	PD	ERP019674	ERS1647278
Bedarf. et al	Parkinson's disease	PD	ERP019674	ERS1647279
Bedarf. et al	Parkinson's disease	PD	ERP019674	ERS1647280
Bedarf. et al	Parkinson's disease	PD	ERP019674	ERS1647281
Bedarf. et al	Parkinson's disease	PD	ERP019674	ERS1647282
Bedarf. et al	Parkinson's disease	PD	ERP019674	ERS1647283
Bedarf. et al	Parkinson's disease	PD	ERP019674	ERS1647284
Bedarf. et al	Parkinson's disease	PD	ERP019674	ERS1647285
Bedarf. et al	Parkinson's disease	PD	ERP019674	ERS1647286
Bedarf. et al	Parkinson's disease	Control	ERP019674	ERS1647303
Bedarf. et al	Parkinson's disease	Control	ERP019674	ERS1647304
Bedarf. et al	Parkinson's disease	Control	ERP019674	ERS1647305
Bedarf. et al	Parkinson's disease	Control	ERP019674	ERS1647306
Bedarf. et al	Parkinson's disease	Control	ERP019674	ERS1647307
Bedarf. et al	Parkinson's disease	Control	ERP019674	ERS1647308
Bedarf. et al	Parkinson's disease	Control	ERP019674	ERS1647309
Bedarf. et al	Parkinson's disease	Control	ERP019674	ERS1647310
Bedarf. et al	Parkinson's disease	Control	ERP019674	ERS1647311
Bedarf. et al	Parkinson's disease	Control	ERP019674	ERS1647312
Milani. et al	Clostridioides difficile infection	CDI	PRJNA297269	SRR2582243
Milani. et al	Clostridioides difficile infection	CDI	PRJNA297269	SRR2582246
Milani. et al	Clostridioides difficile infection	CDI	PRJNA297269	SRR2582247
Milani. et al	Clostridioides difficile infection	CDI	PRJNA297269	SRR2582248
Milani. et al	Clostridioides difficile infection	CDI	PRJNA297269	SRR2582251
Milani. et al	Clostridioides difficile infection	Control	PRJNA297269	SRR2582233
Milani. et al	Clostridioides difficile infection	Control	PRJNA297269	SRR2582234
Milani. et al	Clostridioides difficile infection	Control	PRJNA297269	SRR2582237
Milani. et al	Clostridioides difficile infection	Control	PRJNA297269	SRR2582238
Milani. et al	Clostridioides difficile infection	Control	PRJNA297269	SRR2582241
Tett. et al	Psoriasis (Skin metagenome)	Psoriasis	PRJNA281366	SRR2005538
Tett. et al	Psoriasis (Skin metagenome)	Psoriasis	PRJNA281366	SRR2005673
Tett. et al	Psoriasis (Skin metagenome)	Psoriasis	PRJNA281366	SRR2005659
Tett. et al	Psoriasis (Skin metagenome)	Psoriasis	PRJNA281366	SRR2005707
Tett. et al	Psoriasis (Skin metagenome)	Psoriasis	PRJNA281366	SRR2005712
Tett. et al	Psoriasis (Skin metagenome)	Control	PRJNA281366	SRR2005670
Tett. et al	Psoriasis (Skin metagenome)	Control	PRJNA281366	SRR2005727
Tett. et al	Psoriasis (Skin metagenome)	Control	PRJNA281366	SRR2005657
Tett. et al	Psoriasis (Skin metagenome)	Control	PRJNA281366	SRR2005698
Tett. et al	Psoriasis (Skin metagenome)	Control	PRJNA281366	SRR2005710

Table S2: Comparison of virulence factor prediction with MP3. Evaluated performance of the virulence prediction model versus the MP3 prediction tool regarding sensitivity, specificity and accuracy.

	**MP3**	**PathoFact**
Sensitivity	0.125	0.886
Specificity	0.992	0.957
Accuracy	0.558	0.921

Table S3: Toxin domains differentially abundant in diseased versus control in *Clostridioides difficile* infection.

**HMM Domain**	**Log2Fold Change**	**Name**	**Definition**	**Group**
K11057	-9,61	cpb2	Beta2-toxin	Control
K12788	-6,20	espH	LEE-encoded effector EspH	Control
K01387	-5,94	colA	Microbial collagenase	Control
K11023	-5,01	ptxA, artA	Pertussis toxin subunit 1	Control
PF13945	-4,59	NST1	Salt tolerance down-regulator	Control
PF08998	-4,27	Epsilon antitox	Bacterial epsilon antitoxin	Control
PF15534	-3,83	Ntox35	Bacterial toxin 35	Control
K11062	-3,73	entD	Probable enterotoxin D	Control
K11045	-3,36	cfa	cAMP factor	Control
PF15643	-3,16	Tox-PL-2	Papain fold toxin 2	Control
TIGR03396	-3,10	PC_PLC	Phospholipase C	Control
PF05015	-2,13	HigB-like toxin	RelE-like toxin of type II toxin-antitoxin system HigB	Control
K12340	3,02	tolC	Outer membrane protein	Psoriasis
PF13935	4,70	Ead/Ea22	Ead/Ea22-like protein	Psoriasis
PF14449	4,78	PT-TG	Pre-toxin TG	Psoriasis
K11052	5,20	cylE	CylE protein	Psoriasis

Table S4: Toxin domains differentially abundant in diseased versus control in psoriasis

**HMM Domain**	**Log2Fold Change**	**Name**	**Definition**	**Group**
PF13954	-5,84	PapC_N	PapC N-terminal domain	CDI
PF06609	-3,36	TRI12	Fungal trichothecene efflux pump	CDI
PF13953	-2,90	PapC_C	PapC C-terminal domain	CDI

Table S5: Toxin domains differentially abundant in diseased versus control in Parkinson’s disease.

HMM Domain	Log2Fold Change	Name	Definition	Cohort
K10948	-2.03	hlyA	hemolysin	Control
PF15524	-2.31	Ntox17	Novel toxin 17	Control
PF09599	2.18	IpaC_SipC	Salmonella-Shigella invasion protein c	PD

Table S6: Antimicrobial resistance genes identified within the Klebsiella *pneumoniae* subsp. Pneumoniae HS11286 reference genome.

ARG	ARG_SNPs	Database	Hits
acrA	n/a	DeepARG	1
acrB	n/a	DeepARG	1
acrB	n/a	DeepARG/RGI	1
acrD	n/a	RGI	1
acrF	n/a	DeepARG	1
AcrF	n/a	DeepARG	1
adeB	n/a	DeepARG	1
bacA	n/a	DeepARG	1
bacterial_regulatory_protein_LuxR	n/a	DeepARG	1
baeR	n/a	DeepARG/RGI	1
baeS	n/a	DeepARG	1
bicyclomycin-multidrug_efflux_protein_bcr	n/a	DeepARG	1
CBP-1	n/a	RGI	1
cob(I)alamin_adenolsyltransferase	n/a	DeepARG	1
cpxA	n/a	DeepARG	1
CRP	n/a	DeepARG/RGI	1
DNA-binding_protein_H-NS	n/a	DeepARG	2
emrD	n/a	DeepARG	1
emrR	n/a	DeepARG/RGI	1
eptA	n/a	DeepARG	1
Escherichia coli ampH beta-lactamase	n/a	RGI	1
Escherichia coli EF-Tu mutants conferring resistance to Pulvomycin	R234F	RGI	2
Escherichia coli gyrA conferring resistance to fluoroquinolones	S83I	RGI	1
Escherichia coli marR mutant conferring antibiotic resistance	n/a	RGI	1
Escherichia coli mdfA	n/a	DeepARG/RGI	1
Escherichia coli parC conferring resistance to fluoroquinolone	S80I	RGI	1
Escherichia coli UhpT with mutation conferring resistance to fosfomycin	E350Q	RGI	1
Escherichia_coli_LamB	n/a	DeepARG	1

Escherichia_coli_mipA	n/a	DeepARG	1
FosA6	n/a	DeepARG/RGI	1
Haemophilus influenzae PBP3 conferring resistance to beta-lactam antibiotics	D350N, S357N	RGI	1
kasugamycin_resistance_protein_ksgA	n/a	DeepARG	1
kdpE	n/a	DeepARG	1
Klebsiella pneumoniae acrA	n/a	DeepARG/RGI	1
Klebsiella pneumoniae KpnE	n/a	RGI	1
Klebsiella pneumoniae KpnF	n/a	RGI	1
Klebsiella pneumoniae KpnG	n/a	DeepARG/RGI	1
Klebsiella pneumoniae KpnH	n/a	DeepARG/RGI	1
macA	n/a	DeepARG	1
marA	n/a	DeepARG/RGI	1
mdtB	n/a	RGI	2
mdtC	n/a	RGI	2
mdtD	n/a	DeepARG	1
mdtG	n/a	DeepARG	2
mdtH	n/a	DeepARG	1
mdtK	n/a	DeepARG	4
MdtK	n/a	DeepARG	4
mdtL	n/a	DeepARG	1
mdtM	n/a	DeepARG	2
mdtN	n/a	DeepARG	1
mexX	n/a	DeepARG	1
msbA	n/a	RGI	1
ompF	n/a	DeepARG	2
ompR	n/a	DeepARG	2
patA	n/a	DeepARG	1
PBP-1A	n/a	DeepARG	1
PBP-1B	n/a	DeepARG	1
penA	n/a	DeepARG	2
PmrF	n/a	DeepARG/RGI	1
ramA	n/a	DeepARG	1
rosA	n/a	DeepARG	1
rosB	n/a	DeepARG	1
sdiA	n/a	DeepARG	1
Serratia_marcescens_Omp1	n/a	DeepARG	2
SHV-11	n/a	DeepARG/RGI	1
tet34	n/a	DeepARG	1
TolC	n/a	DeepARG	1
transcriptional_regulatory_protein_CpxR_cpxR	n/a	DeepARG	1
ugd	n/a	DeepARG	1
YojI	n/a	DeepARG	1
